# “We buy what we wanna be”: Understanding the effect of brand identity driven by consumer perceived value in the luxury sector

**DOI:** 10.3389/fpsyg.2022.1002275

**Published:** 2022-09-15

**Authors:** Xi Xi, Jing Yang, Kaiwen Jiao, Shanshan Wang, Tianxiang Lu

**Affiliations:** ^1^Management School, Harbin University of Commerce, Harbin, China; ^2^Zhendao Information Technology Shanghai Co. Ltd., Shanghai, China; ^3^College of Innovative Business and Accountancy, Dhurakij Pundit University, Bangkok, Thailand; ^4^Northeast Branch of CNPC Kunlun Gas Co. Ltd., Dalian, China

**Keywords:** luxury brand, consumer perceived value, social identity, personal identity, brand information quality

## Abstract

Prior studies focused on consumer satisfaction and loyalty have brought undeniable benefits to luxury brand marketing but are not sufficient to ensure a long-lasting and profitable customer-brand relationship in the new setting. Brand identity provides a valuable exploration of this issue. However, the current measurement of brand identity is relatively simple, and there is no clear answer to what factors encourage brand identity development. This study attempts to address this gap by dividing the brand identity structure from a multi-dimensional perspective, considering the role of luxury consumer perceived value and brand information quality in shaping the brand identity. Data was gathered by an online questionnaire survey from Chinese consumers who had purchased luxury jewelry, employing regression methods for analysis. The results show that four predictors representing luxury consumer perceived value all have a significant impact on the brand’s social identity and personal identity. In addition, brand information quality also positively moderates the relationship between the luxury consumer perceived value and the brand’s social identity. This study opens new horizons for considering dimensions other than the satisfaction or intention to use, expanding the applications of brand identity in a new context. The results contribute to increasing the awareness level of brand identity for luxury brand practitioners and offering them a new method of market strategy.

## Introduction

Recently, Colliers published Global Outlook of Luxury Retail in 2022. The report showed that the COVID-19 pandemic caused the blockage or closure of retail stores, reduced international travel, and damaged luxury sales in 2020. The global luxury market declined 12.6% year on year in 2020. However, the gradual resumption of global travel and social activities in 2021 has led to a strong rebound in luxury sales, which increased yearly by 20% to $63.3 billion and surpassed the sales levels in 2020 and 2019. Overall, the consumer’s attitude towards purchasing luxury goods has been rising and has driven a substantial increase in luxury retail sales. Tracing the cause, what makes consumers have a special attachment to luxury brands?

Luxury brands can refer to brands with product attributes such as high quality, high-level aesthetics, and high premiums (Ko et al., 2019). At the same time, it can also be defined as a brand that can bring value attributes such as uniqueness and social status to consumers (Zhang and Zhao, 2019). Research suggests that consumers purchase luxury goods not just to meet basic life needs but for the fact that the symbolic value of luxury brands increases consumers’ self-esteem, the extent of recognition by others, and meets the emotional requirements of individuals (Smith Maguire and Hu, 2013; Li et al., 2022). This can be interpreted as the high quality, high price, and conspicuous image of luxury goods that stimulate consumers’ perceived functional and emotional value, thus creating a deep connection with the brands (Ko et al., 2019).

According to previous research, consumer satisfaction and loyalty are the basic concepts in relationship marketing. Researchers have developed a variety of research frameworks on the relationship between luxury consumer perceived value and the two concepts (Yoo and Park, 2016; Chung and Kim, 2020). However, with time and the change in market competition, the relationship between consumer satisfaction and brand profitability has gradually weakened. The same goes for consumer loyalty. Brand identity refers to consumers’ perceptions, feelings, and evaluations of brand belonging. It helps build sustainable consumer behaviors and promotes stable consumer-brand relationships, which generates high revenue for the brand (Büyükdağ and Kitapci, 2021). Hemonnet-Goujot and Valette-Florence (2022) verified the direct effect of brand identity on luxury brand love and the indirect effect of consumers’ willingness to pay a premium. Gurzki et al. (2019) demonstrated the important role of brand identity for luxury brands, and provided references and suggestions for luxury brands to develop brand identity from the perspective of advertising design and communication planning. These research frameworks confirmed the significant ability of brand identity to meet consumer expectations or differentiate a brand from its competitors. However, previous studies were limited to only taking brand identity as an antecedent without explaining the driving factors behind it. More precisely, little is known about what makes a brand’s identity in existing research, nor is it clear what perceived value consumers value most when purchasing luxury goods.

In addition, we note that the strong consumer demand for luxury brands can be summarized into a two-dimensional framework, including social and personal perceptions (Ma et al., 2021). Social perceptions are related to the status or social impression of luxury brands in social groups. Social identity theory interprets this perception as consumers communicating their social identity to others by using some products (Chan et al., 2015). Personal perceptions are relevant to product quality, hedonic value. Self-congruity theory holds that consumers pursue the unity of perception and personality, behavior, and values (Shetty and Fitzsimmons, 2021). Based on the discussion above, this study argues that the impact of luxury brands on consumers is both social and personal. Compared with a single dimension, a multi-dimensional structure can provide a more comprehensive picture of how a luxury brand identity is created (Shukla et al., 2015). Most studies only regard brand identity as a single-dimensional structure, which lacks multi-level analysis of brand identity (Homburg et al., 2009).

The pleasure and prestige consumer perceived from luxury consumption makes luxury brands highly desired, but brand information also influences consumer behavior (Malik et al., 2013). The popularity of the Internet and the development of social media technology have altered the pattern of brand communication. Brands can communicate with consumers through brand communities, fan pages, and other channels (Jahn and Kunz, 2012; Laroche et al., 2013). Research shows that commodity information provided by luxury brands directly impacts consumers’ perceptions of uniqueness, brand prestige, and artistic appreciation (Kim et al., 2015). Further, it affects consumers’ brand attitudes and purchase intentions (Kong et al., 2021). Some scholars have also proposed that information with higher levels of utilitarian and recreational value will boost consumers’ brand engagement, brand attitude, and purchase intention (Carlson et al., 2008). In this context, the importance of brand information quality is growing (Savolainen, 2011). However, few studies have attempted to examine how brand information quality can influence a brand’s identity, especially within the luxury sector.

In general, existing research analyzes how luxury brands can be better marketed and provides extensive information on the perceived value of luxury brands, but some questions remain to be addressed.

1. What factors motivate consumers to connect deeply with luxury brands?
2. How should we identify the hierarchy of brand identity within the context of luxury sector?
3. Can the information provided by luxury brands support the development of consumer-brand identity?

To fill this research gap, this study sheds light on the significant role of brand identity in shaping consumer-brand relationships and opens new horizons for researchers to consider dimensions of brand identity. Further, in the context of luxury brands, this study identifies consumer perceived value as a key driver of brand identity that has not been fully addressed by prior studies. Besides that, this study investigates the potential moderating effect of brand information quality on the relationship between luxury consumer perceived value and brand identity. The rest of this study is arranged as follows: Section “Theoretical background and hypotheses” reviews the relevant literature on luxury consumer perceived value, brand identity, and brand information quality, from which our hypotheses are formulated. Section “Methodology” describes how the questionnaire survey is carried out and what the results are. Section “Discussion” reports the findings and novelties of this study. Section “Conclusion” summarizes the theoretical contribution, management value, research limitations, and future research.

## Theoretical background and hypotheses

### Brand identity

Brand identity reflects how the brand should be perceived by its target consumers, which emphasizes the psychological connection and loyalty in action between the brand and consumers (Alvarado-Karste and Guzmán, 2020). Different from brand image, which focuses on short-term results, brand identity is the highest level of the consumer–brand relationship and is more strategic (Jin et al., 2019). In this study, brand identity is defined as the unique value perception established by the integration of brand positioning, brand culture, and brand presentation (Jin et al., 2019; Ward et al., 2020). Brand identity communicates the personality and uniqueness of the brand to consumers, gaining their recognition, appreciation, and support (Barros et al., 2020). Therefore, consumers establish a psychological connection with the brand through their value perception of the brand identity, such as conveying an external self-concept and satisfying their internal self-concept. The psychological connection formed between brands and consumers is called consumer-brand identity. It represents the psychological state in which consumers perceive, feel, and value a sense of belonging to a brand (So et al., 2017).

The social (external consistency) needs or the personal (internal consistency) needs influence and stimulate consumers’ behaviors to purchase luxury goods (Alanadoly and Salem, 2021). Social identity theory argues that consumers, influenced by creating and maintaining their social image, are more inclined to choose goods or services that can verify their social identity, and thus develop positive attitudes. Research suggests that luxury consumers’ purchase behavior is an aggregation of consumption behaviors with social value (Reyes-Menendez et al., 2022). Consumers constantly observe the social behavior of reference groups to identify brands that are congruent with their ideal social self-concept (self-image perceived by others) (Sirgy, 1982). By observing and imitating the behavior of reference groups, consumers gain value in terms of a brand’s social identity. A brand’s social identity is at the core of luxury consumption behavior and is a signal of individual wealth and social class (Loureiro et al., 2020). Therefore, consumers enhance their self-image by showing their consumption capacity and expressing their social status through visible evidence of luxury brands (such as brand logos) (O’Cass and McEwen, 2004).

Self-congruity theory states that consumers have their own views on personality, values, and lifestyle. They seek to create a consistent self-concept on the individual side (Sirgy and Su, 2000). Self-consistency represents the tendency of consumers to behave in line with their inner selves. The stronger the perceived consistency, the more likely they are to buy the brand’s products (Ahmad and Thyagaraj, 2014). Donvito et al. (2020) confirmed that personality consistency (prestige, emotion, trust, anxiety, and order) positively affects consumers’ identification of luxury brands. The perception overlap between consumers and brands (namely, brand identity) would promote the consumers’ perceived identity with other groups related to the brand (Carlson et al., 2008).

In conclusion, prior studies support the two types of brand identity (social and personal). Cha et al. (2016) explored the mediating role of brand identity on corporate social responsibility and service brand loyalty from personal and social identity perspectives. Therefore, this study examines the effects of external (social) and internal (personal) needs on consumer purchase behavior toward luxury brands. Furthermore, it describes the formation path of consumer-brand identity for the luxury brand.

### Luxury consumer perceived value

Perceived value is the consumer’s overall assessment of the product’s utility and the perception of personal effort and gain (Chae et al., 2020). In the narrow sense, consumers perceive value when the perceived benefit exceeds the price paid (Sinha and Verma, 2020). Luxury brands can provide consumers with intangible benefits, such as status, uniqueness, and hedonic rewards (Nelissen and Meijers, 2011; Chan et al., 2015; Holmqvist et al., 2020). Previous studies have analyzed the consumer perceptions of luxury brands from different perspectives. These studies can be summarized into two categories: personal-oriented and social-oriented perceived values (Wiedmann et al., 2009). The personal-oriented perceived values of luxury brands emphasize the functional and financial aspects perceived by consumers, which mainly involve price, economic value, and quality (Sheth et al., 1991). The social-oriented perceived values of luxury brands focus on the driving force of consumers to create an excellent social image (Sallot, 2002). That is, the idea that consumers expect to “impress others” by wearing or using luxury goods.

Sweeney and Soutar (2001) combined the two perspectives and measured consumer perceived value in multiple dimensions, including price, functional, emotional, and social value. It has become a classic marketing method and is widely employed in follow-up research. For example, Li et al. (2012) divided luxury consumer perceived value into three types: emotional, utilitarian, and economic value, analyzing their impact on consumers’ purchase intention. Loureiro et al. (2020) further expanded the research on luxury consumption behaviors, evaluating consumers’ perceptions of internal self-concept and social self-concept from four dimensions: personal, social, functional, and economic value. So it seems that consumer perceived value has been viewed as a multi-dimensional structure in luxury marketing. Therefore, this study utilizes the measurement developed by Sweeney and Soutar (2001) to measure luxury consumer perceived value across four dimensions: social value, emotional value, functional value, and economic value. This strategy aims to construct a comprehensive framework for evaluating the consumer perceived value of luxury brands, thereby providing research ideas applicable to the luxury industry.

#### Luxury consumer perceived social value

Social value derives from the ability of a product to enhance social self-concept (Sweeney and Soutar, 2001). Consumers may associate psychological clues with products or incorporate brands’ symbolic meanings into their identities (Vigneron and Johnson, 2004). In this sense, consumers build their social identity by purchasing or possessing luxury goods, classifying themselves as members of ideal social groups to attain social values such as desirable prestige, status, or social image. Shukla et al. (2015) pointed out that consumers’ attitudes toward luxury brands depend on their social perception of luxury brands. The current research supports the idea that luxury brands have conspicuous (attractive) and prestige value that can help consumers gain acceptance within their social groups (Wiedmann et al., 2007). Therefore, it can be inferred that luxury consumer perceived social value positively affects the brand’s social identity.

In addition to social identity, the self-congruity theory explains that consumers seek consistency in cognition (such as beliefs, values, and personality) and behavior. Inconsistency would make consumers feel worried, anxious, or dissatisfied (Wang et al., 2022). Thus, consumers are more likely to have positive attitudes and behaviors when they hold consistent (similar) beliefs about objects or events (Sirgy et al., 1997). A sense of luxury will lead to a high degree of inner self-consistency for consumers (O’Cass and Lim, 2002). Through a study of luxury fashion accessories, Liu et al. (2012) confirmed that personality congruity positively influences consumer loyalty toward luxury brands. Therefore, it is reasonable to assume that the luxury consumer perceived social value positively affects the brand’s personal identity. The following hypotheses are formulated in this part:

H1: Luxury consumer perceived social value positively relates to the brand’s social identity.

H2: Luxury consumer perceived social value positively relates to the brand’s personal identity.

#### Luxury consumer perceived emotional value

Besides social value, brands provide consumers with experiences, feelings, and emotions. Emotional value describes the subjective utility and intrinsically pleasing attributes that consumers acquire from purchasing or owning products, and the emotions or emotional states they derive (evoke) from them (Wuestefeld et al., 2012). Stokburger-Sauer et al. (2012) confirmed that emotion-related brand experiences could help build consumer-brand identification. Feelings such as “luxury brands make me happy” and “luxury brands make me feel good” influence consumers’ emotional attitudes toward luxury brands (Sweeney and Soutar, 2001). Therefore, luxury can offer consumers a high level of external sensory satisfaction and conspicuous value.

Moreover, the perceived personality consistency between brands and individuals plays an important role in consumers’ connection with brands based on emotional commitment (Stokburger-Sauer et al., 2012). The expression ability of luxury brands to convey personality reinforces consumers’ emotional return on luxury brands and motivates consumers to show their characteristics through luxury consumption (Bian and Forsythe, 2012).

Therefore, consumers’ emotional attitudes toward a luxury brand can affect the brand’s ability to meet consumers’ social needs and express their personalities. The following hypotheses are formulated in this part:

H3: Luxury consumer perceived emotional value positively relates to the brand’s social identity.

H4: Luxury consumer perceived emotional value positively relates to the brand’s personal identity.

#### Luxury consumer perceived functional value

Functional value refers to what consumers expect from a luxury brand in terms of superior product quality (including product availability, reliability, and durability), as well as unique services (Sheth et al., 1991; Wiedmann et al., 2007). Consumers often associate luxury brands with superior goods and services, which means they expect more value (Aaker, 2009). Studies have demonstrated that luxury consumption results in superior goods and assists consumers in advancing their social standing (Li et al., 2022). The social identity theory explains that consumers’ self-improvement and self-esteem through brands, which are important sources of a brand’s social identity (Bhattacharya and Sen, 2003). Therefore, it can be concluded that the high-quality attributes of luxury goods can enhance the brand’s social identity.

The matching of brand functionality with consumer expectations or relevant evaluation criteria is called functional consistency (Su and Reynolds, 2017). Functional consistency affects consumer attitudes toward brands, as consumers are always more inclined to products that can represent their values (Lee and Jeong, 2014). Moreover, when the brand image matches the consumer’s internal self-image, the emotional bond between consumers and the brand is strengthened (Evanschitzky and Wunderlich, 2006). The impressive design and excellent quality of luxury goods are both the source of conspicuous consumption and an extension of consumers’ internal self-image (Jacob et al., 2020). As a result, it is believed that the consumer-perceived functional consistency on luxury goods can boost a brand’s personal identity. The following hypotheses are formulated in this part:

H5: Luxury consumer perceived functional value positively relates to the brand’s social identity.

H6: Luxury consumer perceived functional value positively relates to the brand’s personal identity.

#### Luxury consumer perceived economic value

Economic value involves monetary aspects such as prices, discounts, and investments. It can also be expressed as the opportunity cost that consumers pay for luxury goods (Wiedmann et al., 2007). Luxury goods are often expensive, and consumers are accustomed to associating unique, high-quality products with high prices (Heine and Phan, 2011). In particular, luxury goods are generally considered to be of social prestige by social-oriented consumers. Consumers believe that the more expensive a luxury item is, the more valuable it is and the more worthwhile it is to buy (Hennigs et al., 2013). This provides an answer to why consumers are willing to pay a premium price for luxury goods. Because expensive luxury goods become a symbol of consumers’ wealth, reputation, and social status, making consumers willing to pay higher prices (Balabanis and Stathopoulou, 2021).

The consistency of values explains the perceived similarity between the values held by individuals and organizations. When individual values are consistent with the brand’s, consumers’ psychological results (i.e., trust, satisfaction, and positive behavioral intention) tend to be enhanced (Lee and Jeong, 2014). Park et al. (2008) argued that consumers purchase luxury goods because the symbolic meaning of such products corresponds to their values, such as exclusivity and aesthetics. In other words, the high price of luxury goods conveys the intrinsic value of luxury brand holders, which will affect their brand preference and willingness to pay a premium price for the brand. Therefore, under the effect of economic value perception, consumers create meaningful brand associations with luxury brands through image matching. The following hypotheses are formulated in this part:

H7: Luxury consumer perceived economic value positively relates to the brand’s social identity.

H8: Luxury consumer perceived economic value positively relates to the brand’s personal identity.

### Brand information quality

Information quality refers to the quality level of helpful information obtained by members of the community (Lin et al., 2019), which can be measured in terms of accuracy, completeness, clarity, understandability, usefulness, and reliability (Chen and Chang, 2018). Luxury brand marketing must communicate to consumers a clear brand position, exclusivity, and other supporting descriptions (Fionda and Moore, 2009). Previous research has confirmed that high-quality information can have an incremental impact on consumers’ willingness to interact with luxury brands. Specifically, high-quality information denotes the ability to make consumers perceive the relevance of content, trigger consumer brand associations, help consumers convey information about their status, prestige, social achievement, and reflect the brand’s well-designed, superior-quality products (Bazi et al., 2020).

Among them, the perceived relevance of the content reflects consumers’ perception of the brand’s social value. The brand creates a prominent social image and communicates it to consumers in a timely manner, which can prompt consumers to match the brand image with their external image and shape consumer brand attitudes (Kim and Ryu, 2021). Therefore, brand information quality will affect consumers’ perception of brand image and help them judge whether such perception conforms to their external social image.

Besides that, consumers often anthropomorphize or personalize various objects when making purchasing decisions (Sirgy, 1982; Chen and Lin, 2021). Brand information provides consumers with clues to evaluate the brand. High-quality information support can help consumers capture useful information, strengthen the connection between consumers’ self-concept and brand image, and influence consumers’ brand choice intentions. Based on the above discussion, the following hypotheses can be established:

H9: Brand information quality moderates the relationship between luxury consumer perceived social value and the brand’s social identity.

H10: Brand information quality moderates the relationship between luxury consumer perceived social value and the brand’s personal identity.

The ability of brand association is related to consumers’ emotional attributes. Social interaction with luxury brands is also a form of self-fulfillment for consumers, enhancing their identification and emotional engagement (Bazi et al., 2020). For example, timely information push service, thoughtful page designs, and helpful informational content all make customers feel special and create an emotional connection with the brand. Therefore, consumers will get a good sense of social value when luxury brands provide accurate, timely, and reliable information support (Kim et al., 2012). In this case, consumers will be more willing to engage with the brand and other consumers. And their enthusiasm for the brand and sense of social identity will be enhanced (Lin et al., 2019).

Besides that, consumers can learn more about luxury brands through platform interaction and other channels, enhance their emotional attachment to the brand and make more informed consumption decisions. When the information provided by luxury brands is more related to consumers’ internal characteristics, consumers’ personal identification with the brand increases (Shahid and Paul, 2021). Based on the above discussion, the following hypotheses can be established:

H11: Brand information quality moderates the relationship between luxury consumer perceived functional value and the brand’s social identity.

H12: Brand information quality moderates the relationship between luxury consumer perceived emotional value and the brand’s personal identity.

Functional value reflects the essential utility of luxury goods and is one of the reasons why consumers choose luxury goods (Zhang and Zhao, 2019). Brand attributes such as usability, uniqueness, and quality are communicated to consumers through advertising, social media, and other information exchanges, which can significantly influence consumers’ purchasing decisions. Among them, social-oriented consumers pay more attention to the symbolic value of luxury goods, including the values of showing off and conformity. Such consumers seek to connect with desirable groups through outstanding product design and quality, or to differentiate themselves from undesirable groups (Ostovan and Nasr, 2022).

Personal-oriented consumers pay more attention to the practical value of luxury goods than social-oriented. Such consumers emphasize the superior capacity of luxury goods to convey intrinsic value, which involves satisfying the expression of their inner self (Jebarajakirthy and Das, 2021). Based on the above discussion, the following hypotheses can be established:

H13: Brand information quality moderates the relationship between luxury consumer perceived functional value and the brand’s social identity.

H14: Brand information quality moderates the relationship between luxury consumers’ perceived functional value and the brand’s personal identity.

Luxury goods are usually expensive compared to necessities. Thus, the consumption of luxury goods often implies the level of wealth from which an individual’s status and power can be inferred (Shahid and Paul, 2021). High-quality information can help consumers obtain useful information, encourage consumers to make changes in behavior and cognition, and improve consumers’ willingness to pay (Elsharnouby et al., 2021). Driven by social-oriented strong desires such as vanity and social status, consumers hope that the use of luxury goods will help them form and change social identities, defining whom they want to be.

In addition, luxury brands give information that the brand and the target consumers are highly similar in attitude, behavior, and values through relevant media, which makes the target consumers and the brand have an emotional connection (Liu et al., 2018; Loureiro et al., 2020). Driven by such internal demands, consumers argue that the cost of acquiring luxury goods is worthwhile and have positive perceptions and emotions towards the brand. Based on the above discussion, the following hypotheses can be established:

H15: Brand information quality moderates the relationship between luxury consumer perceived economic value and the brand’s social identity.

H16: Brand information quality moderates the relationship between luxury consumer perceived economic value and the brand’s personal identity.

**FIGURE 1:**
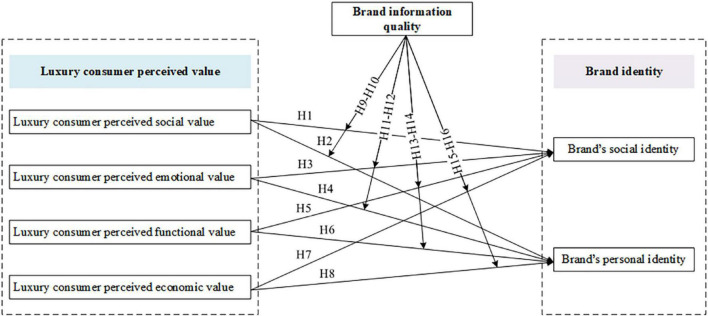
Conceptual framework.

According to the above hypotheses, the conceptual framework is summarized in Figure 1.

## Methodology

### Data collection and analysis

Jewelry is an essential luxury category, representing global brands such as Cartier, BVLGARI, and Van Cleef & Arpels. Deloitte published Global Powers of Luxury Goods in 2020, which included some statistics. According to the number of companies ranked on the list, the jewelry companies occupied the second most. There are nine Chinese companies listed among them, all of which are jewelry companies. This shows that the jewelry industry is very representative as a research object for luxury marketing research. Therefore, this study selected luxury jewelry as the research object.

A questionnaire was designed for this study to collect data, and the questionnaire was composed of four parts. The first part discusses the respondents’ characteristics (including gender, age, occupation, and consumption status). The second part demonstrates consumers’ perceived value of luxury jewelry brands, including consumers’ perceived social, emotional, functional, and economic value. The third part represents the brand identity of luxury jewelry, which includes social identity and personal identity. The fourth part outlines the moderating effect of brand information quality on luxury consumer perceived value-brand identity. The questionnaire contains a total of 29 items that are graded on the Likert 5-point scale: “1 means strongly disagree; 2 means disagree; 3 means generally agree; 4 means somewhat agree; 5 means strongly agree.” Table 1 shows the specific constructs and related references. First, we assess luxury consumer perceived value with 16 items based on the scale design of Sweeney and Soutar (2001). The measurement of a brand’s social identity refers to Bhattacharya and Sen (2003), which includes five items. Brand’s personal identity refers to Del Rio et al. (2001), which contains four items. Finally, this study summarized the measurement methods by Lee et al. (2002), which used four questions to assess the quality of brand information.

**TABLE 1:** Constructs and references related to scales.

Constructs	References of the scales
Luxury consumer perceived social value	Sweeney and Soutar, 2001
Luxury consumer perceived emotional value	
Luxury consumer perceived functional value	
Luxury consumer perceived economic value	
Brand’s social identity	Bhattacharya and Sen, 2003
Brand’s personal identity	Del Rio et al., 2001
Brand information quality	Lee et al., 2002

This study aims to analyze the role of luxury consumer perceived value in shaping luxury brands’ identities. To enhance the sample representativeness, this study selected effective samples that meet the research objectives by setting up constraints. Firstly, adults over 18 years of age were taken as the target population. Secondly, only consumers with luxury jewelry buying experience were invited to participate in the survey (Loureiro et al., 2020). This study collected questionnaires by publishing the QR code and website link of the questionnaire through social software such as WeChat, QQ, and the community. The respondents were anonymous and voluntarily participated in the survey. The survey won’t share the private information of the people who fill it out, and the data it gets is only used for academic research, not for commercial purposes.

A total of 314 questionnaires were received. After eliminating the questionnaires that did not meet the requirements, such as the answering time being too short, the selected answers being basically the same, or the answers being presented regularly and incomplete, 204 valid questionnaires were finally obtained. The characteristics of the respondents are shown in Table 2. Among them, 88 (43.10%) are male and 116 (56.90%) are female. The ratio of males to females is basically balanced. Besides that, most respondents are aged 25–31, with a bachelor’s degree and a monthly living cost of between 5,001 and 8,000 yuan RMB.

**TABLE 2:** Demographics of the respondents.

Demographics	Frequency	Percentage
**Gender**		
Male	88	43.10%
Female	116	56.90%
**Age**		
18–24	34	16.67%
25–31	98	48.04%
32–38	55	26.96%
Over 39	17	8.33%
**Educational background**		
Senior high school or below	14	6.86%
College	33	16.18%
Undergraduate	118	57.84%
Graduate school or above	39	19.12%
**Average monthly living expenses**		
Less than CNY 1,000	7	3.43%
Between CNY 1,001 and less than CNY 3,000	24	11.76%
Between CNY 3,001 and less than CNY 5,000	46	22.55%
Between CNY 5,001 and less than CNY 8,000	68	33.33%
Greater than CNY 8,001	59	28.92%

Data source: questionnaire survey.

### Reliability and validity analysis

Following the conceptual framework, this study employs several procedures to examine the impact of predictors on the brand identity of luxury brands. The specific data analysis process includes: In the first stage, we evaluated the reliability and validity of the model. Then, we adopted the regression analysis method to verify the research hypotheses in the second stage. Results details are described below.

As shown in Table 3, the Cronbach’s alpha value of all variables was higher than 0.7, which was in line with the strong reliability standard (0.7) proposed by Nunnally and Bernstein (1994), indicating that the questionnaire had good reliability.

**TABLE 3:** Assessment of reliability and convergent validity.

Scale items	Estimate	AVE	C.R.
**Luxury consumer perceived social value (Cronbach’s α = 0.898)**			
Buying the brand’s jewelry has raised my social status.	0.829	0.689	0.899
Wearing the brand’s jewelry makes me feel accepted by others in a situation.	0.798		
Wearing the brand’s jewelry helps to make a good impression.	0.827		
Wearing the brand’s jewelry can improve how others see me.	0.865		
**Luxury consumer perceived emotional value (Cronbach’s α = 0.836)**			
The brand’s jewelry makes me feel relaxed.	0.762	0.508	0.837
The brand’s jewelry will make me happy.	0.768		
The brand’s jewelry gives me self-satisfaction.	0.676		
The brand’s jewelry is my favorite.	0.718		
The brand’s jewelry is something I want to wear every day.	0.631		
**Luxury consumer perceived functional value (Cronbach’s α = 0.798)**			
The brand’s jewelry is of good quality.	0.820	0.591	0.812
The brand’s jewelry is an exquisite craft.	0.749		
The brand’s jewelry lasts a long time.	0.734		
**Luxury consumer perceived economic value (Cronbach’s α = 0.801)**			
The brand’s jewelry is reasonably priced.	0.749	0.511	0.806
The brand’s jewelry offers value that is worth the price.	0.688		
The brand’s jewelry is a good item for its price.	0.759		
The cost of time and effort was worth it for the value the brand’s jewelry provided.	0.658		
**Brand’s social identity (Cronbach’s α = 0.858)**			
Buying this brand’s jewelry reflects my social status.	0.718	0.561	0.864
Buying this brand’s jewelry will give me respect from others.	0.818		
Buying this brand’s jewelry will give me social recognition.	0.781		
Buying this brand’s jewelry helps me separate from other groups of people.	0.617		
Buying this brand’s jewelry makes it easier for me to get recognized.	0.793		
**Brand’s personal identity (Cronbach’s α = 0.822)**			
The brand’s jewelry matches my personalized pursuits.	0.733	0.538	0.823
The brand’s jewelry matches my values.	0.778		
The brand’s jewelry is in line with my personal taste.	0.687		
The brand’s jewelry fits my lifestyle.	0.732		
**Brand information quality (Cronbach’s α = 0.823)**			
The information published by the brand is normative.	0.743	0.539	0.824
The information published by the brand is time-sensitive.	0.696		
The information published by the brand is novel.	0.781		
The brand’s messages are diverse.	0.714		

Data source: questionnaire survey.

Three measures [average variance extracted (AVE), composite reliability (CR), and correlation coefficient matrix] are used to test the validity of the questionnaire. As shown in Table 3, the AVE ranged from 0.508 to 0.689, which was higher than the recommended value of 0.5, and the C.R. was 0.806 to 0.899, which was greater than the threshold value of 0.7, indicating that the scale items had good convergent validity (Fornell and Larcker, 1981; Hair et al., 2006). Therefore, the scale items in this study had good convergent validity. The internal consistency of each variable was good, and it had convergent validity in all cases.

As seen in Table 4, all constructs matched the discriminant validity standard, as the square roots of the AVE were greater than the correlations between construct pairs (Fornell and Larcker, 1981). Therefore, it can be concluded that this questionnaire had good discriminant validity.

**TABLE 4:** Assessment of discriminant validity.

Variables	F1	F2	F3	F4	F5	F6	F7
Luxury consumer perceived social value	**0.830**						
Luxury consumer perceived emotional value	0.575^***^	**0.713**					
Luxury consumer perceived functional value	0.343^***^	0.671^***^	**0.769**				
Luxury consumer perceived economic value	0.328^***^	0.498^***^	0.588^***^	**0.715**			
Brand’s social identity	0.671^***^	0.426^***^	0.413^***^	0.453^***^	**0.749**		
Brand’s personal identity	0.436^***^	0.637^***^	0.555^***^	0.467^***^	0.563^***^	**0.733**	
Brand information quality	0.363^***^	0.494^***^	0.535^***^	0.366^***^	0.386^***^	0.644^***^	**0.734**

The bold values are the square root of the average variance extracted. ^***^p < 0.001. Data source: questionnaire survey.

This study also tested the ability of the measurement model to fit the observed data. As shown in Table 5, χ^2^/*df* = 1.489, RMSEA = 0.049, CFI = 0.940, TLI = 0.932, IFI = 0.941, all fit indices matched the recommended levels, showing that the model had a good fitting ability (Hu and Bentler, 1999).

**TABLE 5:** Model fit coefficients.

Indices	χ^2^/*df*	RMSEA	IFI	TLI	CFI
Value	1.489	0.049	0.940	0.932	0.941
Cut-off point	≤5	<0.1	≥0.9	≥0.9	≥0.9

Data source: questionnaire survey.

Furthermore, this study tested whether the questionnaire had a common method bias. According to factor analysis, six factors were extracted, and the contribution rate of the largest factor was 33.828%, which was conformed to the critical standard. This suggested that the effect of common method bias was not significant and that the data could be used for hypotheses analysis.

### Hypothesis analysis

#### Tests of the direct effect hypotheses

The effect of four attributes (social, emotional, functional, and economic) of the luxury consumer perceived value on the brand identity (social identity and personal identity) was examined. Table 6 shows the regression results of the hypotheses test. As expected, all of the four predictors show significant effects, indicating that the luxury consumer perceived value has a significant positive impact on brand identity. Luxury consumer perceived social, functional, emotional, and economic value positively influences the brand’s social identity. Among them, the social value shows a highly significant positive effect (β = 0.520, *p* < 0.001), followed by the economic value (β = 0.486, *p* < 0.001). Luxury consumer perceived social, functional, emotional, and economic value are positively related to the brand’s personal identity. Among them, emotional value is the strongest predictor (β = 0.510, *p* < 0.001), followed by the functional value (β = 0.453, *p* < 0.001). The test results of the constructs towards the brand’s personal identity are different from social identity. Luxury consumer perceived social value and economic value are more effective for the brand’s social identity, while emotional value and functional value are more important for the brand’s personal identity.

**TABLE 6:** Hypotheses testing results.

Hypotheses	Independent variable	Dependent variable	Coefficient	Conclusion
**The direct effect-social identity**				
H1	Luxury consumer perceived social value	Brand’s social identity	0.520***	Supported
H3	Luxury consumer perceived emotional value	Brand’s social identity	0.388***	Supported
H5	Luxury consumer perceived functional value	Brand’s social identity	0.407***	Supported
H7	Luxury consumer perceived economic value	Brand’s social identity	0.486***	Supported
**The direct effect-personal identity**				
H2	Luxury consumer perceived social value	Brand’s personal identity	0.296***	Supported
H4	Luxury consumer perceived emotional value	Brand’s personal identity	0.510***	Supported
H6	Luxury consumer perceived functional value	Brand’s personal identity	0.453***	Supported
H8	Luxury consumer perceived economic value	Brand’s personal identity	0.435***	Supported
**The moderating effect-social identity**				
H9	Luxury consumer perceived social value	Brand’s social identity	0.257***	Supported
H11	Luxury consumer perceived emotional value	Brand’s social identity	0.156*	Supported
H13	Luxury consumer perceived functional value	Brand’s social identity	0.334***	Supported
H15	Luxury consumer perceived economic value	Brand’s social identity	0.086	Not Supported
**The moderating effect-personal identity**				
H10	Luxury consumer perceived social value	Brand’s personal identity	0.009	Not Supported
H12	Luxury consumer perceived emotional value	Brand’s personal identity	–0.041	Not Supported
H14	Luxury consumer perceived functional value	Brand’s personal identity	0.107	Not Supported
H16	Luxury consumer perceived economic value	Brand’s personal identity	–0.140	Not Supported

*p < 0.1 and ^***^p < 0.001. Data source: questionnaire survey.

#### Tests of the moderation effect hypotheses

This study adopts hierarchical regression analysis to test the moderating effect. Firstly, the interaction term is obtained by multiplying the luxury consumer perceived value and the brand information quality. Then, the relationship between interaction items and brand identity is introduced and tested in the hierarchical regression test. If the regression result of the interaction term reaches a significant level, the moderating effect is supported.

A moderated analysis with brand information quality as the moderator is performed. As shown in Table 6, three of four moderating hypotheses towards brand’s social identity are supported, namely H9, H11, and H13. Details are as follows:

The information quality positively moderates the effect of luxury consumer perceived social value and the brand’s social identity (β = 0.257, *p* < 0.001), suggesting that social value’s impact on the brand’s social identity becomes more prominent when the brand information is considered as high quality. Hence, H9 is supported. Brand information quality strengthens the luxury consumer perceived emotional value and the brand’s social identity linkages (β = 0.156, *p* < 0.1), indicating that emotional value’s impact on the brand’s social identity becomes stronger for high-quality brand information than for low-quality brand information. Hence, H11 is supported. Furthermore, the effect of luxury consumer perceived functional value on the brand’s social identity (β = 0.334, *p* < 0.001) is also positively moderated by brand information quality, showing that the better functional perception a brand provides to consumers, the stronger the consumer’s connection to brand’s social identity. Hence, H13 is supported. The moderation effect of brand information quality on the luxury consumer perceived economic value and the brand’s social identity is statistically insignificant (β = 0.086), showing that price information provided by the brand has no significant effect on the luxury consumer perceived emotional value and the brand’s social identity connections.

Next, the moderating effect of brand information quality on luxury consumer perceived value and brand’s personal identity is performed. According to the hierarchical regression analysis, we can’t find a significant influence for the brand information quality, indicating that the brand information quality cannot moderate the effect of luxury consumer perceived value on brand’s personal identity.

## Discussion

Brand identity can maximize consumer value, help brands stand out, and gain solid consumer-brand relationships, which are the key to brand survival. This study explores the influence of luxury consumer perceived value, an antecedent variable, on establishing brand identity and the moderating role of brand information quality. The contributions made by this study can be reflected from the following three aspects.

### Double identity of luxury brands

This study distinguishes the double identity of luxury brands, namely social identity and personal identity. Unlike other marketing concepts, brand identity is seen as the ultimate expression of the consumer-brand connection. Past research on luxury relationship marketing has treated brand identity as a one-dimensional structure, which limited the in-depth discussion on consumers’ purchasing motives for luxury brands. Therefore, based on social identity theory and self-congruity theory, this study divides brand identity into a two-dimensional structure that includes both social and personal identity. This multi-dimensional structure broadens the understanding of brand identity in luxury relationship marketing research.

### Effects of luxury consumer perceived value on brand identity

This study investigates the different effects of luxury consumer perceived value multi-dimensions in forming the two brand identities. Past research has focused on how luxury consumer perceived value evokes positive emotional responses to luxury brands. We infer that luxury consumer perceived value is a multi-dimensional construct, and not all dimensions are equally relevant to brand identity. The specific analysis is as follows:

#### Brand’s social identity as the outcome

This study examines the role of luxury consumer perceived value in developing a brand’s social identity. The results confirm that the four factors representing the luxury consumer perceived value all have a significant positive relationship with the brand’s social identity. Among them, social value played the most important role. This result is not difficult to understand, given that social value is the central component of luxury goods and a crucial motivator of consumers’ purchasing behavior (Wiedmann et al., 2007; Kastanakis and Balabanis, 2014; Bazi et al., 2020). Social value determines consumers’ attitudes and perceptions of luxury brands (Shukla et al., 2015). This result is supported by Sheth et al. (1991). That is, consumers are motivated to purchase gold products by social value to gain the recognition of their ideal group. In this case, other values, such as functional and emotional value, only have a minor influence on purchase behavior (Sheth et al., 1991). Economic value is another strong predictor. Consumers tend to associate high prices with superior quality and uniqueness (Reyes-Menendez et al., 2022). Even if some luxury goods are less cost-effective, they provide consumers with intangible and contextual utility (Wiedmann et al., 2007). This makes consumers feel that the effort and sacrifice are worthwhile, thereby inspiring consumers to identify with the power of luxury brands such as conspicuous.

However, the results are in contrast to Reyes-Menendez et al. (2022), in which consumers’ economic motivation for purchasing luxury goods no longer matters. Research suggests that consumer purchasing behavior may also depend on the consumer environment, especially market maturity (Siahtiri and Lee, 2019). Reyes-Menendez et al. (2022) studied luxury consumers in Madrid, Spain. Studies have shown that wealthy consumers in mature markets, as part of the social elite, place more value on their inner self (self-expression). They do not expect to differentiate themselves from others through luxury goods, but value the functional attributes of luxury goods, such as durability and quality (Pino et al., 2019). Consumers in emerging markets see luxury goods as a visible symbol of wealth. They tend to show their status in society and the reference groups to which they belong through conspicuous consumption (Zici et al., 2021). High prices, noticeable appearance, and a sense of luxury become important clues to show off one’s wealth and status (Shahid and Paul, 2021). This study focuses on luxury jewelry consumers in China, so the economic value perceived by consumers still plays an important role.

#### Brand’s personal identity as the outcome

This study analyzes the role of luxury consumer perceived value in developing a brand’s personal identity. All hypotheses about luxury consumer perceived value and the brand’s personal identity are also supported. Nevertheless, in contrast to the brand’s social identity, emotional value and functional value play an important role. Emotional value primarily provides consumers with intangible benefits such as sensory and emotional attachment to satisfy their inner self-definition (Ajitha and Sivakumar, 2017). Luxury goods’ appearance and functional expression trigger consumers’ hedonic value and encourage consumers to form brand impressions (Hemonnet-Goujot and Valette-Florence, 2022).

Why do luxury consumer perceived value play diametrically opposite roles in shaping two brand identities? Similar to brand identity, luxury consumer perceived value can also be classified as other-oriented and self-oriented. The perceived value of other-oriented includes others’ impressions, self-expectations, and gaining others’ respect concerning property (Tynan et al., 2010; Han and Kim, 2020). The self-oriented perceived value consists of the functional value (performance, superior quality) and emotional value (self-value, aesthetics, perfectionism) (Tynan et al., 2010; Han and Kim, 2020). Therefore, considering luxury consumer perceived value as the predictor of brand identity, other-oriented value (i.e., social value and economic value) has a fitting advantage among the regression coefficients of the brand’s social identity. Similarly, self-oriented value (i.e., functional value and emotional value) is advantageous for the brand’s personal identity.

### Moderating role of brand information quality

This study explores the moderating effect of brand information quality. Marketing research on luxury brands has always focused on consumer behaviors without considering changes in the external market environment. However, consumers’ new lifestyles and consumption behaviors drive the evolution of the luxury market. Therefore, this study incorporates the quality of brand information provided by luxury brands into the research framework as a moderating variable. The results found that brand information quality can moderate the relationship between perceived value and the brand’s social identity among luxury consumers. This is consistent with the test result of the direct effect. Thus, what consumers expect most from luxury brands is still the social benefits. Among the regression coefficients of moderating effect, luxury consumer perceived functional and social value play the most prominent roles. This implies that luxury brands should provide more sensory symbols, such as conspicuous design, superior quality, etc., to enhance consumers’ perceptions of their social status and self-esteem (Noble and Kumar, 2010). These actions can increase consumers’ enthusiasm for and identity with the brand.

## Conclusion

### Theoretical contribution

This study provides valuable theoretical contributions to the literature on the consumer-brand relationship in luxury marketing by taking the luxury jewelry consumer as a case study. Firstly, this study develops an integrated framework in which luxury consumer perceived value is the antecedent and brand identity is the outcome. Such a relationship appears to have not been empirically validated in the literature on luxury brands. Further, this study regards brand identity as a two-dimensional variable, including social identity and personal identity. Compared with single-dimensional, multi-dimensional division aids in clarifying and understanding the driving outcomes of different factors on brand identity, which are rarely considered by other studies.

Secondly, this study compares the different roles of luxury consumer perceived value in different dimensions in developing two brand identities. Existing studies have consistently agreed on the powerful role of brand identity, but there is still insufficient research on what drives the establishment of brand identity. Therefore, this study treats luxury consumer perceived value as the antecedent of brand identity to construct a theoretical model. From the perspective of consumers’ internal and external perceptions, identify which factor is the most critical to developing the luxury brand’s identity. The results show that the other-oriented luxury consumer perceived value is more conducive to developing the brand’s social identity. In contrast, the self-oriented perceived value is more favorable to formulating the brand’s personal identity. The results provide an effective reference for consumer psychology and luxury consumption studies.

Thirdly, brand information quality provides decision support for consumers and influences consumers’ purchase intentions. Consumers’ motivations for purchasing luxury goods differ from those for purchasing ordinary products. Therefore, previous marketing research may not be adaptable to the particularity of luxury brand management. Combined with the consumers’ anticipated value for luxury goods, this study highlights the features of luxury brand information, such as reliability, uniqueness, and relevance, to analyze its moderating effect on brand identity development. The results show that brand information quality effectively develops the luxury brand’s social identity. Consumers place a premium on brand information corresponding to luxury goods’ social, functional, and emotional value. This provides new concerns for research on consumer-brand relationships in the luxury sector.

### Management value

This study provides a clear and comprehensive explanation of the mechanism between luxury consumer perceived value and brand identity, offering recommendations for luxury brands to implement effective marketing strategies.

Firstly, perceived value drives consumers to satisfy one or more self-defined needs, view the luxury brand as an integral part of their identity, and establish a more durable and stable association with luxury brands. The results show that luxury consumer perceived social, emotional, functional, and economic values positively affect the brand’s social identity and personal identity. The point is that luxury consumer perceived social and economic value mainly affect the brand’s social identity, while emotional and functional value primarily affect the brand’s personal identity. Luxury brands can develop marketing strategies by understanding this dichotomy, combining luxury consumer perceived value with the potential of brand identity to spark consumer enthusiasm. Brands can design some limited, unique, superior products for consumers that favor social value. Create a perception of scarcity for them and release a signal to improve their social image. For example, designing brand logos that are noticeable and easily observed by consumers. For consumers that place a premium on personal identity, brands should focus on matching consumers’ inner self-image through products, services, and marketing experiences. Giving products human characteristics, or associating the brand with consumers’ personality traits, can enhance their awareness of the brand’s ability to express an inner self-concept. Brands should balance the ability to express both identities.

Secondly, the quality of brand information provided by luxury brands affects the strength of consumer perceived value on brand identity. Enhancing the level of information quality would positively help form a meaningful consumer-brand identity. Therefore, it’s highly recommended that luxury brands integrate communication strategies with consumer perceived value, consider how they may better express brands, and differentiate themselves. Timely and useful information support can help consumers easily get more relevant information about the products or services they are interested in. Moreover, it can lead consumers to perceive a higher level of social support and significantly enhance brand recognition, especially when consumers precisely need the product. Therefore, luxury brands can update brand information timely to provide consumers with accurate and relevant brand information to meet their social, hedonic, functional, and other perceived needs. In addition, the results emphasize the important role of brand information quality in shaping a brand’s social identity. Therefore, luxury brand marketing could focus more on the aspects related to social-oriented value, convincing consumers that they can achieve social reputation and influence others’ opinions through brands.

### Limitations and future research

This study makes a worthwhile attempt to test the impact of luxury consumer perceived value on brand identity. As with any study, this study has several limitations that may provide the opportunity for further research. First, the sample size is relatively small and is currently in its exploratory phase. In the future, we can attempt to obtain larger samples for research, maybe the results of this study will be further strengthened. Second, this study does not consider the characteristics of consumers, such as cultural background differences. In the future, we can investigate the impact of cultural differences on consumers’ desires to purchase luxury goods, such as individualism and collectivism. Third, this study chooses the jewelry category as the research object of luxury consumer-brand relationships. The particularities of different categories, such as clothing, leather goods, cosmetics, and automobiles, are not included in the research scope, which also indicates the future direction of luxury marketing research.

## Data availability statement

The raw data supporting the conclusions of this article will be made available by the authors, without undue reservation.

## Author contributions

XX proposed the idea and design of the study. JY was responsible for the analysis and interpretation of the data, as well as completing the article for this study. KJ contributed to the data collection and cleaning. SW served as research advisor. TL edited and revised the manuscript together with others. All authors contributed to the article and approved the submitted version.
